# HER2-intronic miR-4728-5p facilitates HER2 expression and accelerates cell proliferation and migration by targeting EBP1 in breast cancer

**DOI:** 10.1371/journal.pone.0245832

**Published:** 2021-02-02

**Authors:** Yu Zhou, Yuan Yuan, Liuyi Li, Xueliang Wang, Yimin Quan, Chunyu Liu, Mengchao Yu, Xiuting Hu, Xiangfeng Meng, Zhen Zhou, Chen-Yu Zhang, Xi Chen, Minghui Liu, Chen Wang

**Affiliations:** Nanjing Drum Tower Hospital Center of Molecular Diagnostic and Therapy, Chinese Academy of Medical Sciences Research Unit of Extracellular RNA, State Key Laboratory of Pharmaceutical Biotechnology, Jiangsu Engineering Research Center for MicroRNA Biology and Biotechnology, NJU Advanced Institute for Life Sciences (NAILS), Institute of Artificial Intelligence Biomedicine, School of Life Sciences, Nanjing University, Nanjing, Jiangsu, China; University of Ulsan College of Medicine, REPUBLIC OF KOREA

## Abstract

HER2 amplification greatly contributes to the tumorigenesis of multiple cancers. Intronic miR-4728-5p is transcribed along with its host gene HER2. However, little is known about the role of miR-4728-5p in cancer. This study aims to elucidate the potential role of miR-4728-5p and the underlying mechanism in breast cancer. Kaplan-Meier analysis showed that higher expression of HER2 led to worse survival outcomes in breast cancer patients. The TCGA dataset revealed that compared to normal breast tissues, HER2 and miR-4728-5p levels were significantly upregulated in breast cancer tissues with a positive correlation. In functional assays, miR-4728-5p was confirmed to promote the proliferation and migration in breast cancer cell BT474. EBP1 was identified as a direct target of miR-4728-5p via bioinformatics and luciferase reporter assays. miR-4728-5p was further demonstrated to increase HER2 expression and promote cell proliferation and migration by directly inhibiting EBP1 in breast cancer. Taken together, the HER2-intronic miR-4728-5p/EBP1/HER2 feedback loop plays an important role in promoting breast cancer cell proliferation and migration. Our study provides novel insights for targeted therapies of breast cancer.

## Introduction

Breast cancer is the most common malignant tumor diagnosed among women, accounting for 30% of all new cancer cases in United States this year [1]. Despite many advances in the diagnosis and treatment over the past years, more than 42,260 women in United States are estimated to die of breast cancer this year [2]. Hence, it is of great significance to investigate the breast cancer pathogenesis and explore novel potential therapeutic targets for treatment.

microRNAs (miRNAs) are single-stranded noncoding RNA molecules of approximately 19–25 nucleotides and ubiquitously expressed in all kinds of tissues. miRNAs directly target the 3’ untranslated regions (3’UTR) of message RNAs (mRNAs) by complementary base pairing, leading to silencing or degradation of targeted mRNAs [3]. miRNA dysregulations are crucially involved in diverse human diseases including cancer [4]. These dysregulated miRNAs play oncogenic or tumor-suppressive roles in the development and progression of cancer by participating in various cellular activities such as proliferation, differentiation, apoptosis, migration and invasion [3, 4].

Intronic miRNAs are derived from the introns of protein-coding genes and transcribed along with their host genes [5, 6]. miR-4728 is a new-identified intronic miRNA derived from HER2 (erb-b2 receptor tyrosine kinase 2, also known as ErbB2) [7, 8], which belongs to the epidermal growth factor receptor (EGFR) family. As a well-known oncogene, HER2 was found to be amplified in around 25% of breast cancers [9]. HER2 amplification was highly associated with the recurrence and disease-related death of breast cancer [10, 11]. In recent years, some groups also raised concern about HER2-encoded miR-4728 in the cancer research field. For example, miR-4728-3p was reported to target ESR1 (Estrogen Receptor 1) via a non-canonical seed interaction [7]; another report further demonstrated that co-amplification of miR-4278 could protect HER2-positive breast cancers from lapatinib treatment through repressing ESR1 [12]. Besides, miR-4728-3p could participate in regulating the 3’ tailing and trimming of miR-21-5p through suppressing poly(A) RNA polymerase D5 (PAPD5) [13]. However, so far, the function of miR-4728-5p has not been fully investigated yet.

In this study, we demonstrated HER2-encoded miR-4728-5p could augment cell proliferation and migration by directly targeting ErbB3-binding protein 1 (EBP1) in breast cancer. Moreover, miR-4728-5p-mediated downregulation of EBP1 in turn enhanced HER2 expression, indicating a feedback loop consisting of HER2, miR4728-5p and EBP1. Our study suggests miR-4728-5p as a novel potential therapeutic target and provides new insights for the tumorigenesis in breast cancer.

## Materials and methods

### Cells

Breast cancer cells MCF-7 (HER2-) and BT474 (HER2+) were purchased from Shanghai Institute of Cell Biology, Chinese Academy of Sciences (Shanghai, China) and cultured under suggested conditions [12]. MCF-7 cells were cultured with the high glucose Dulbecco’s modified eagle medium (high glucose DMEM, Gibco, USA) supplemented with 10% fetal bovine serum (FBS, Gibco, USA). BT474 cells were grown in RPMI-1640 medium (Gibco, USA) supplemented with 10% FBS. Cells were maintained at 37°C in humidified conditions containing 5% CO2.

### RNA isolation and quantitative RT-PCR

Total RNA was extracted using the TRIzol (Sigma, USA). miRNA and mRNA quantifications were performed respectively using Taqman probes (Applied Biosystems, USA) or SYBR Green Dye (Invitrogen, USA) as previously shown [14]. U6 and GAPDH served as internal controls. Primers sequences were as follows: EBP1 forward: 5’-AGCGACCAGGATTATATTCTCAAG-3’; EBP1 reverse: 5’-ATAACATCTGCTTTCCTCCCTG-3’; HER2 forward: 5’-AGCCTTGCCCCATCAACTG-3’; HER2 reverse: 5’-AATGCCAACCACCGCAGA-3’; GAPDH forward: 5’-GCACCGTCAAGGCTGAGAAC-3’; GAPDH reverse: 5’- TGGTGAAGACGCCAGTGGA -3’.

### Western blot

Total protein was isolated using RIPA Lysis buffer (Beyotime, China). Protein concentration was quantified with a Pierce BCA kit (Thermo Scientific, USA). Samples were separated by 10% SDS-PAGE gel (Bio-Rad, USA). Antibodies against EBP1 (sc-393114), HER2 (sc-33684) and GAPDH (sc-166574) were purchased from Santa Cruz Biotechnology.

### Luciferase reporter assay

The EBP1 3’UTR fragment containing miR-4728-5p binding site was inserted into the pMIR-reporter vector (Ambion, USA). The wild-type binding site was mutated from CCCUCCC to GGGAGGG in order to generate a mutant luciferase vector. 293T cells were utilized to perform the luciferase report assay. miR-4728-5p mimics or inhibitor was transfected using Lipofectamine 2000 (Invitrogen, USA) along with luciferase vectors and β-gal control plasmid. The fluorescence value was assayed using a luciferase assay kit (Promega, USA). Relative luciferase activity was normalized to β-gal.

### Plasmid construction and siRNA interference assay

The full-length open reading frame of human EBP1 was cloned into a mammalian expression plasmid (Invitrogen, USA). An empty plasmid served as the negative control. EBP1 siRNA (sense: 5’-GTGAGGTGGAAAGGCGT-3’) was synthesized by GenePharma (China). The EBP1 expression plasmid and siRNA were transfected using Lipofectamine 2000 (Invitrogen, USA). Total RNA and protein were isolated 24 h post-transfection. Protein and mRNA levels were assessed by Western blot and quantitative RT-PCR.

### Cell proliferation and migration assays

BT474 cells were transfected and seeded into 96-well plates (6×10^3^ cells per well) for the proliferation activity which was determined at 0, 6, 12, 18, and 24 h post-transfection using the Cell Counting Kit-8 (Dojindo, Japan) according to the manufacturer’s instructions. For the migration assay, transfected BT474 cells were treated with mitomycin C (10μg/ml, R&D Systems, USA) for 10 h to prevent proliferation, and equal number of cells were then seeded into the 24-Well Millicell plates (Millipore, Germany) containing an 8-μm pore membrane as previously described [14] and the transwell-containing plates were incubated for 24 h.

### Bioinformatic and statistical analysis

Targetscan [15] was used to predict potential targets of miR-4728-5p. The TCGA data portal was used to explore the HER2 and miR-4728-5p levels and their correlation in breast cancer tissues. Kaplan-Meier curves were utilized to generate the overall survival (OS) outcomes of breast cancer patients (http://kmplot.com/analysis/). Experiments were repeated in triplicate. Student’s t-test was applied to analyze differences between two groups. P values were considered to be statistically significant when it is less than 0.05.

## Results

### High HER2 and miR-4728 levels lead to poor survival outcomes of breast cancer patients and miR-4728-5p is upregulated in breast cancer tissues along with HER2

**Fig 1:**
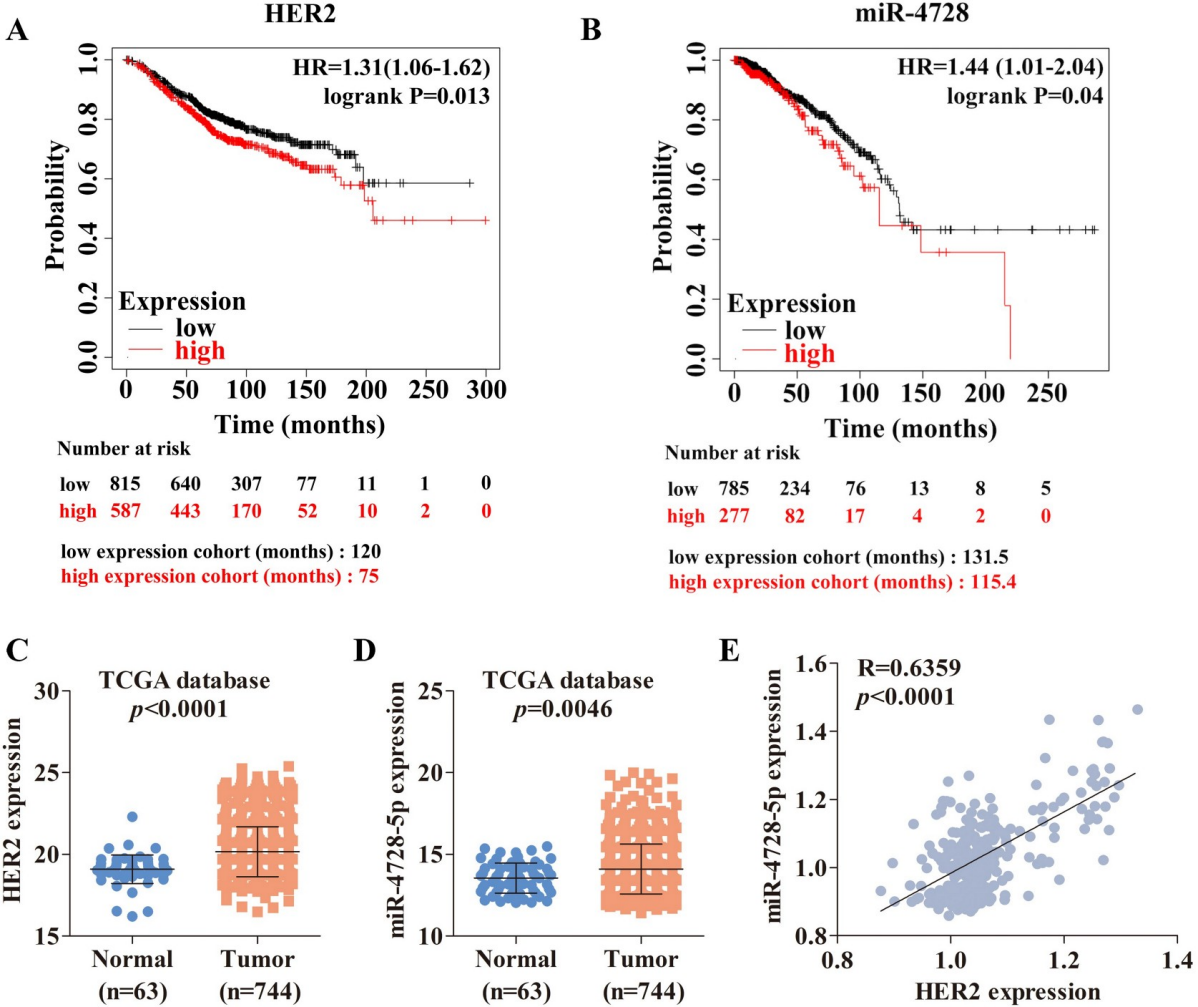
The association of survival outcomes in breast cancer patients with HER2 expression and miR-4728-5p levels in breast cancer tissues based on the TCGA dataset. (A and B) Influence of HER2 and miR-4728 expression on overall survival (OS) by Kaplan-Meier analysis in breast cancer patients. The detailed information for the analysis of survival outcomes with HER2 (mRNA database [16]) and miR-4728 (miRNA database [17]) expression was in S1 Table. (C and D) Analyses of HER2 and miR-4728-5p expression levels in normal breast tissues and breast cancer tissues based on the TCGA dataset. (E) The Pearson’s correlation analysis between the expression levels of miR-4728-5p and HER2 in breast cancer tissues (BRCA) from the TCGA dataset (S2 Table).

We assessed the survival outcomes of breast cancer patients using Kaplan-Meier curves (http://kmplot.com/analysis/) and found that higher-expressed HER2 (Fig 1A) or higher-expressed miR-4728 (Fig 1B) resulted in worse overall survival (OS). Next, we determined the high expressions of HER2 (Fig 1C) and miR-4728-5p (Fig 1D) in breast cancer patients based on the TCGA dataset. miR-4728-5p was found to be positively correlated with HER2 expression (Fig 1E). In addition, this positive correlation also exists in Lung Adenocarcinoma (LUAD) and Stomach adenocarcinoma (STAD) (S1 Fig).

### miR-4728-5p promotes breast cancer cell proliferation and migration

**Fig 2:**
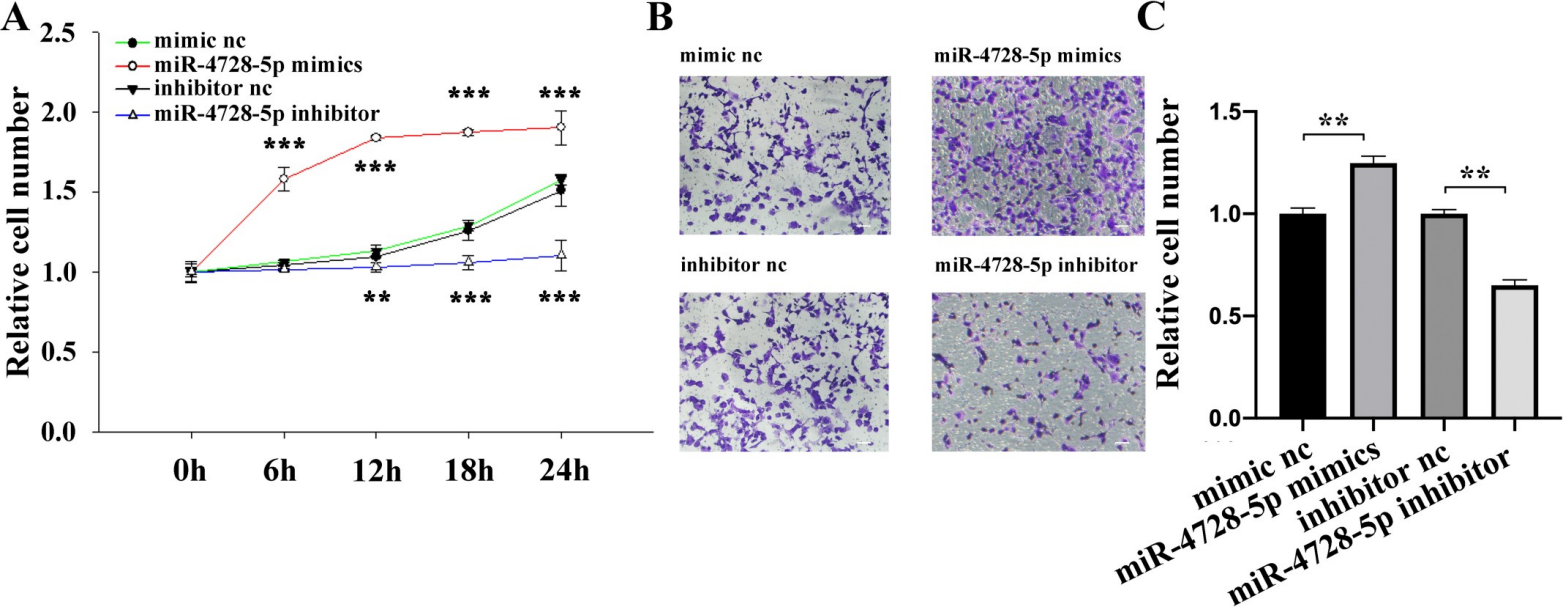
miR-4728-5p plays an oncogenic role in breast cancer cells. (A) Overexpression of miR-4728-5p remarkably promoted the proliferation activity of BT474 cells and inhibition of miR-4728-5p reduced cell proliferation. (B and C) The migratory ability of BT474 cells was enhanced by miR-4728-5p overexpression but repressed by miR-4728-5p inhibition. Scale bar, 50μm. **p<0.01, ***p<0.001.

The biological function of miR-4728-5p was studied in BT474 cell using CCK-8 (Cell Counting Kit-8) and transwell assays. As shown, overexpression of miR-4728-5p remarkably promoted cell proliferation (Fig 2A) and migration (Fig 2B and 2C) and vice versa, indicating that miR-4728-5p functions as an oncogene in the breast cancer tumorigenesis.

### EBP1 is directly downregulated by miR-4728-5p, which in return promotes HER2 expression

**Fig 3:**
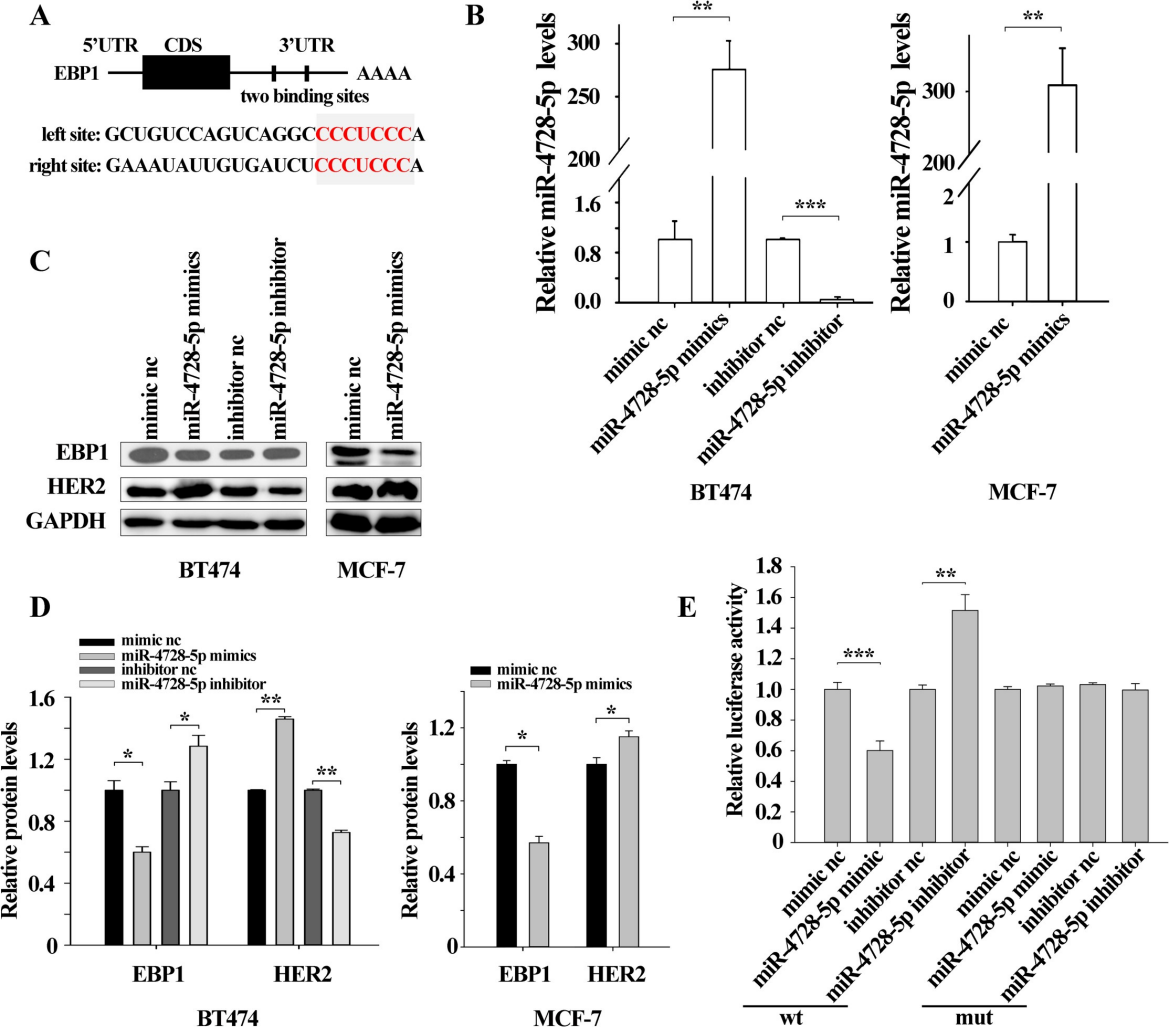
EBP1 is directly inhibited by miR-4728-5p. (A) The schematic diagram of the predicted binding sites of miR-4728-5p at the EBP1 3’ UTR. (B) miR-4728-5p expression levels were successfully increased by miR-4728-5p mimics and decreased by miR-4728-5p inhibitor. (C and D) miR-4728-5p overexpression lessened EBP1 protein levels and enhanced HER2 protein levels. Conversely, miR-4728-5p downregulation increased EBP1 protein expressions and inhibited HER2 protein expressions. (E) Firefly luciferase reporters containing either wild-type (wt) or mutant (mut) miR-4728-5p binding sites in the EBP1 3’-UTR were co-transfected with equal doses of miR-4728-5p mimic, miR-4728-5p inhibitor or the ncRNA. Relative luciferase activity was markedly reduced in the cells transfected with miR-4728-5p mimic and increased in those transfected with miR-4728-5p inhibitor. The mutated luciferase report was unaffected by either overexpression or knockdown of miR-4728-5p. *p<0.05, **p<0.01, ***p<0.001.

Among the potential targets of miR-4728-5p predicted via bioinformatics, EBP1 drew our attention as it could suppress HER2 transcription by binding to the promoter region [18]. Two predicted binding sites of miR-4728-5p in the 3’UTR of EBP1 were illustrated (Fig 3A). To explore the exact relationship between miR-4728-5p and EBP1, we overexpressed or knocked down miR-4728-5p using miR-4728-5p mimics or inhibitor both in BT474 and MCF-7 cells (Fig 3B). Due to the low expression level of miR-4728-5p in MCF-7 (S2 Fig), knockdown of miR-4728-5p was not performed in MCF-7 cells. Western blot data showed that miR-4728-5p overexpression dramatically downregulated EBP1 protein levels while miR-4728-5p knockdown remarkably increased EBP1 protein levels (Fig 3C and 3D). Because EBP1 was a negative regulator of HER2, here we also detected HER2 protein levels. As shown, HER2 protein levels were enhanced by miR-4728-5p overexpression but decreased by miR-4728-5p knockdown (Fig 3C and 3D). Luciferase reporter assays further confirmed the direct binding between miR-4728-5p and EBP1 3’UTR. The fluorescence intensity was weakened by miR-4728-5p overexpression but strengthened by miR-4728-5p knockdown (Fig 3E).

### miR-4728-5p accelerates cell proliferation and migration by targeting EBP1

**Fig 4:**
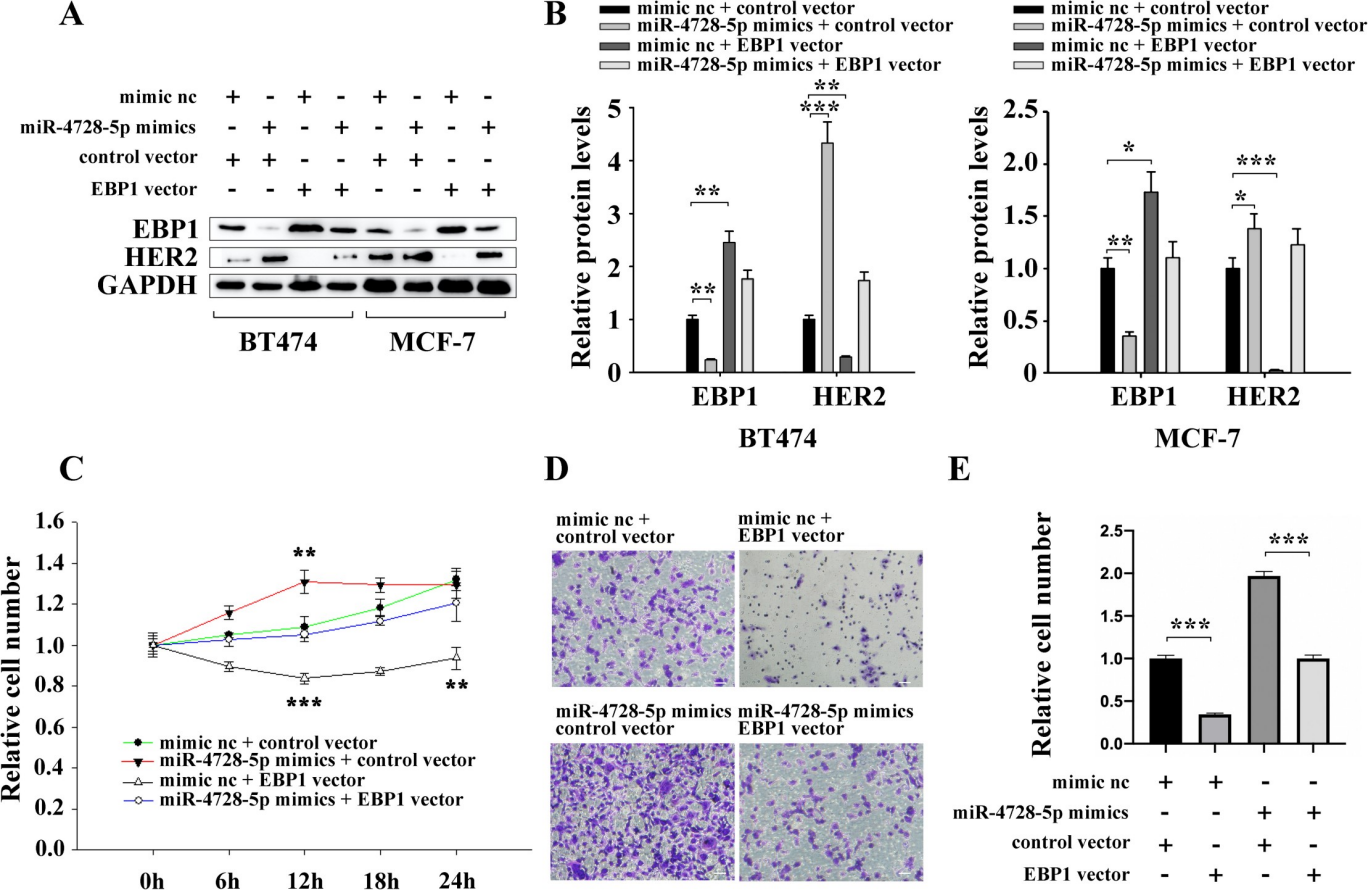
miR-4728-5p promotes breast cancer cell proliferation and migration by suppressing EBP1. (A and B) EBP1 vector successfully restored the EBP1 protein levels that were reduced by miR-4728-5p, while the change in HER2 protein expression was just the opposite of EBP1. (C) miR-4728-5p facilitated BT474 cell proliferation by inhibiting EBP1. (D and E) miR-4728-5p promoted BT474 cell migration by inhibiting EBP1. Scale bar, 50μm. **p<0.01, ***p<0.001.

To figure out whether miR-4728-5p promotes cell proliferation and migration by targeting EBP1, we constructed an EBP1 overexpression vector without the miR-4728-5p responsive 3’UTR to specifically restore EBP1 expression inhibited by miR-4728-5p. Subsequently, restoration of EBP1 (Fig 4A and 4B) completely abolished the promotion effects of miR-4728-5p on cell proliferation and migration (Fig 4C–4E). These results suggest that miR-4728-5p is an oncogenic miRNA that accelerates breast cancer cell proliferation and migration by directly suppressing EBP1.

## Discussion

The EGFR family, crucially involved in the tumorigenesis of multiple cancers, comprises four receptors, namely, EGFR/HER1, HER2, HER3 and HER4. Dimerization between EGFR members is essentially required for the signaling activities and functions of these receptors [19]. For example, HER2 is a transmembrane tyrosine kinase without a known ligand while HER3 lacks innate kinase activity [20]. However, HER2 can heterodimerize with HER3 to trigger a complex network of key signaling pathways including the MAPK and PI3K-Akt pathways, resulting in cell proliferation, survival, division and motility [20, 21]. The HER2-HER3 dimer is considered as the most active EGFR signaling dimer and is crucial for the activation of downstream signaling pathways in tumors with HER2 amplification [22]. In addition, co-amplification of HER2 and HER3 is frequently found in human malignant tumors [23–25] and is remarkably associated with the poor prognosis of breast cancer [25].

EBP1 is a ligand of HER3 but can reduce HER2 transcriptions by mitigating the activity of HER2 promoter [26]. Studies have reported that EBP1 could inhibit cell growth in breast cancer [27] and increase the sensitivity of prostate cancer cells to lapatinib [28]. However, the regulatory mechanism of EBP1 in breast cancer remains not fully clarified.

In the present study, we first focused on the potential function of HER2-encoded miR-4728-5p in breast cancer. Through analyzing gene expressions from a TCGA cohort of breast cancer patients, we found miR-4728-5p was highly expressed along with HER2 in the cancer tissues. Pearson analysis showed a positive correlation between miR-4728-5p and HER2 expression levels. Furthermore, overexpressing of miR-4728-5p dramatically accelerated the proliferation and migration activities of BT474 cells, while silencing of miR-4728-5p had the reverse effect, indicating miR-4728-5p plays an oncogenic role in breast cancer.

When further predicting the potential target genes of miR-4728-5p using bioinformatics, we found EBP1 that had two binding sites of miR-4728-5p at the 3’UTR. We next tried to figure out whether HER2-encoded miR-4728-5p could directly regulate EBP1. The luciferase reporter assays verified the direct binding between miR-4728-5p and EBP1 3’UTR. Overexpression of miR-4728-5p significantly repressed EBP1 protein expressions as well as increased HER2 protein levels in BT474 and MCF-7 cells. We also introduced the rescue experiments to confirm the negative regulation of EBP1 by miR-4728-5p. EBP1 overexpression vector was used to specifically restore EBP1 expression inhibited by miR-4728-5p. As a result, restoration of EBP1 in BT474 completely abolished the promoting effect of miR4728-5p on cell proliferation and migration.

Based on these results, we draw an intriguing conclusion that HER2-intronic miR-4728-5p could facilitate the expression of its host gene HER2 by directly repressing EBP1. Thus, co-amplification of HER2 and miR-4728-5p plays a dual oncogenic role in the pathogenesis of breast cancer. The feedback loop of HER2-intronic miR-4728-5p/EBP1/HER2 is of great importance in HER2-positive breast cancer and leads to poor prognosis. Our findings may provide a better understanding of HER2 signaling pathway in breast cancer, as well as potential therapeutic targets.

## Supporting information

S1 Fig The positive correlation between miR-4728-5p and HER2 expression in Lung Adenocarcinoma (LUAD) and Stomach adenocarcinoma (STAD) tissues based on the TCGA dataset. (A and B) The Pearson’s correlation analysis between the expression levels of miR-4728-5p and HER2 in LUAD and STAD tissue obtained from the TCGA dataset (S2 Table).
(TIF)

S2 Fig The RNA expression levels in MCF-7 and BT474. The RNA expression levels of miR-4728-5p, EBP1 and Erbb2 in MCF-7 and BT474. The expression levels of miR-4728-5p and Erbb2 mRNA are very low. ***p<0.001.
(TIF)

S3 Fig Uncropped Western blot image for Fig 3C. (TIF)

S4 Fig Uncropped Western blot image for Fig 4A. (TIF)

S1 Table The detailed information for the analysis of survival outcomes with HER2 and miR-4728 expression. (DOCX)

S2 Table The original analyzed data of gene expressions based on the TCGA dataset. (XLS)
