# Supplementary figures and images for: HER2-intronic miR-4728-5p facilitates HER2 expression and accelerates cell proliferation and migration by targeting EBP1 in breast cancer
Source: PLoS One. 2021 Feb 2;16(2):e0245832. doi: 10.1371/journal.pone.0245832 (PMC7853520; doi:10.1371/journal.pone.0245832)

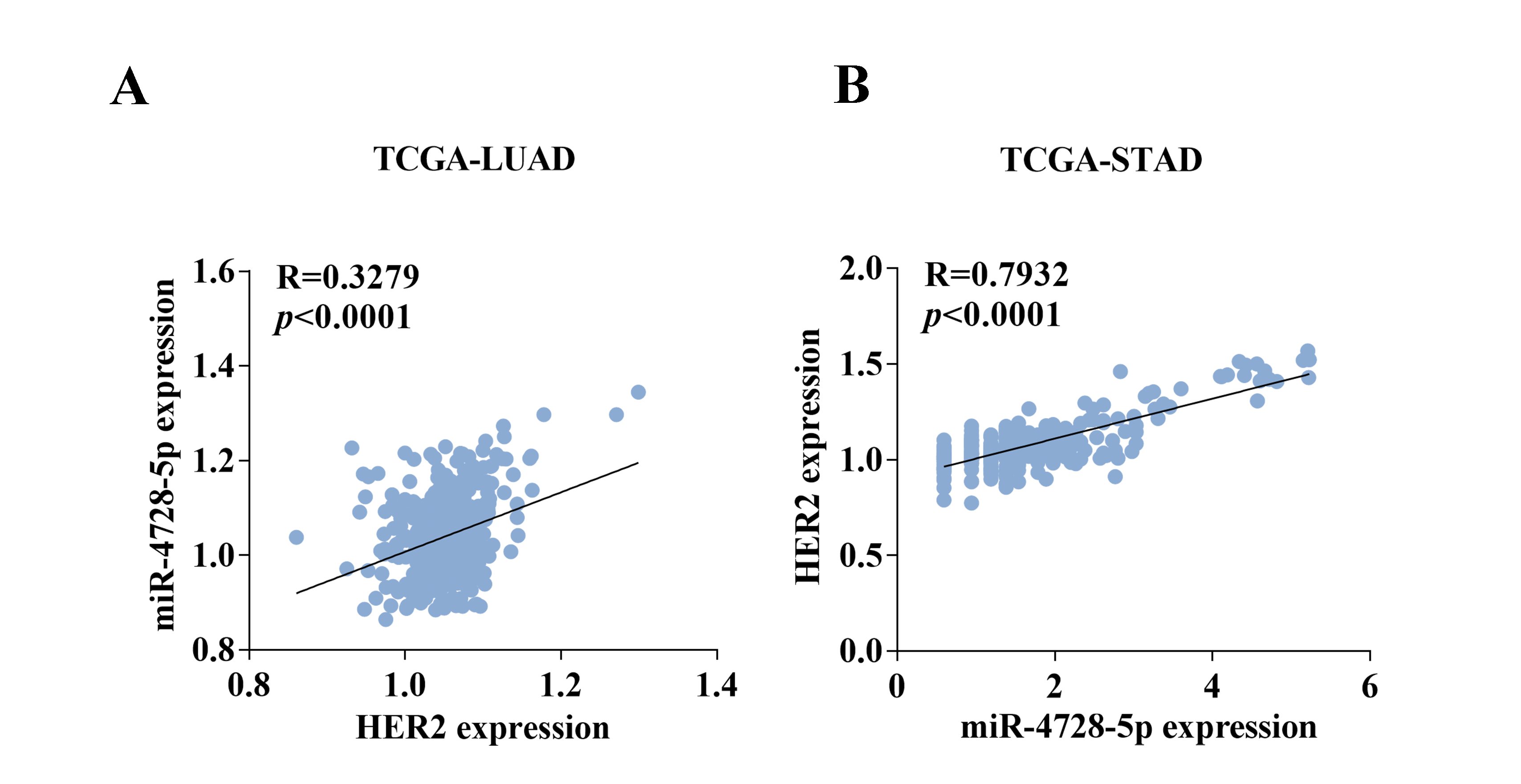

Supplement: S1 Fig — (A and B) The Pearson’s correlation analysis between the expression levels of miR-4728-5p and HER2 in LUAD and STAD tissue obtained from the TCGA dataset (S2 Table). (TIF) [file pone.0245832.s001.tif]

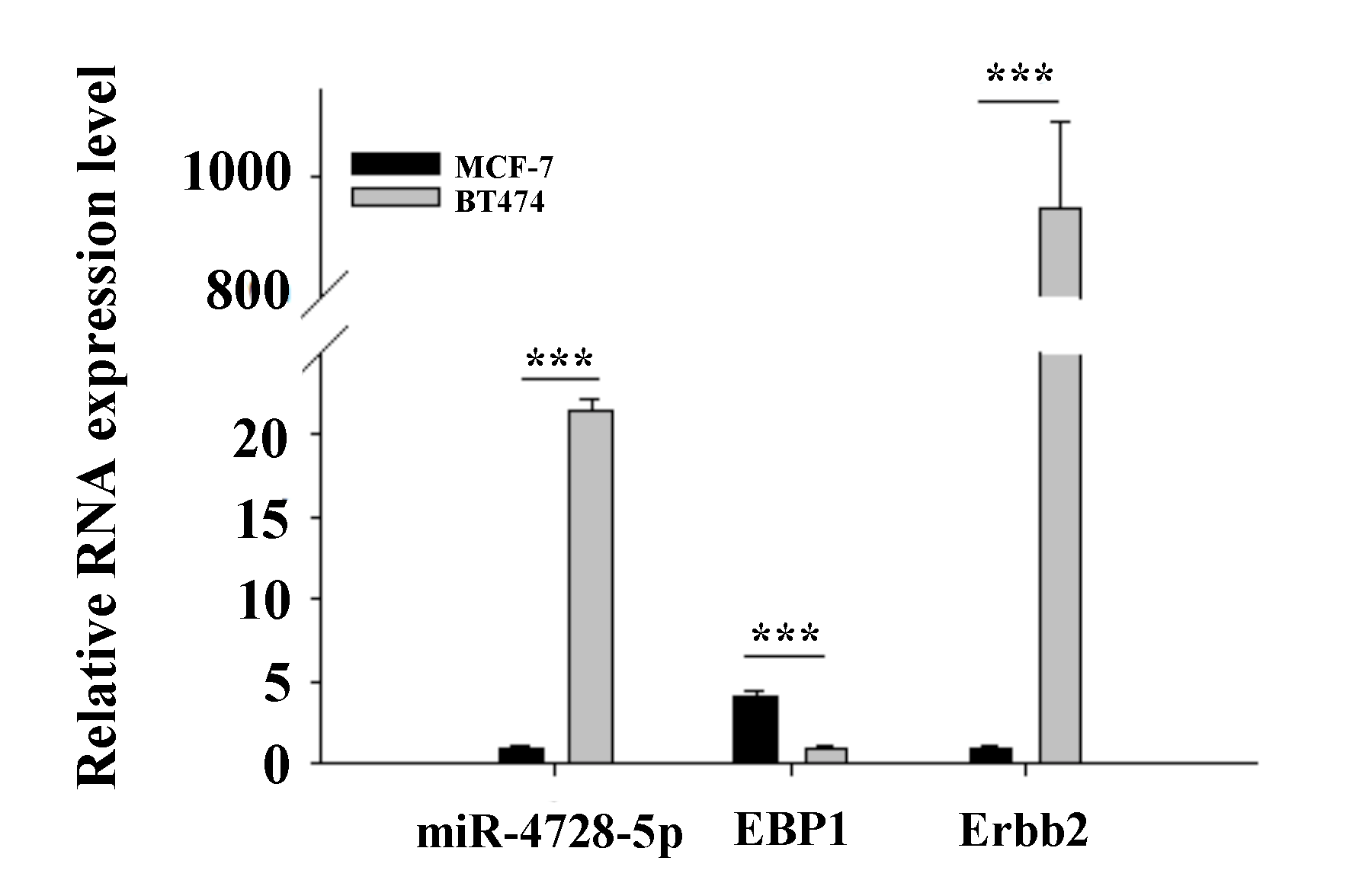

Supplement: S2 Fig — The RNA expression levels of miR-4728-5p, EBP1 and Erbb2 in MCF-7 and BT474. The expression levels of miR-4728-5p and Erbb2 mRNA are very low. ***p<0.001. (TIF) [file pone.0245832.s002.tif]

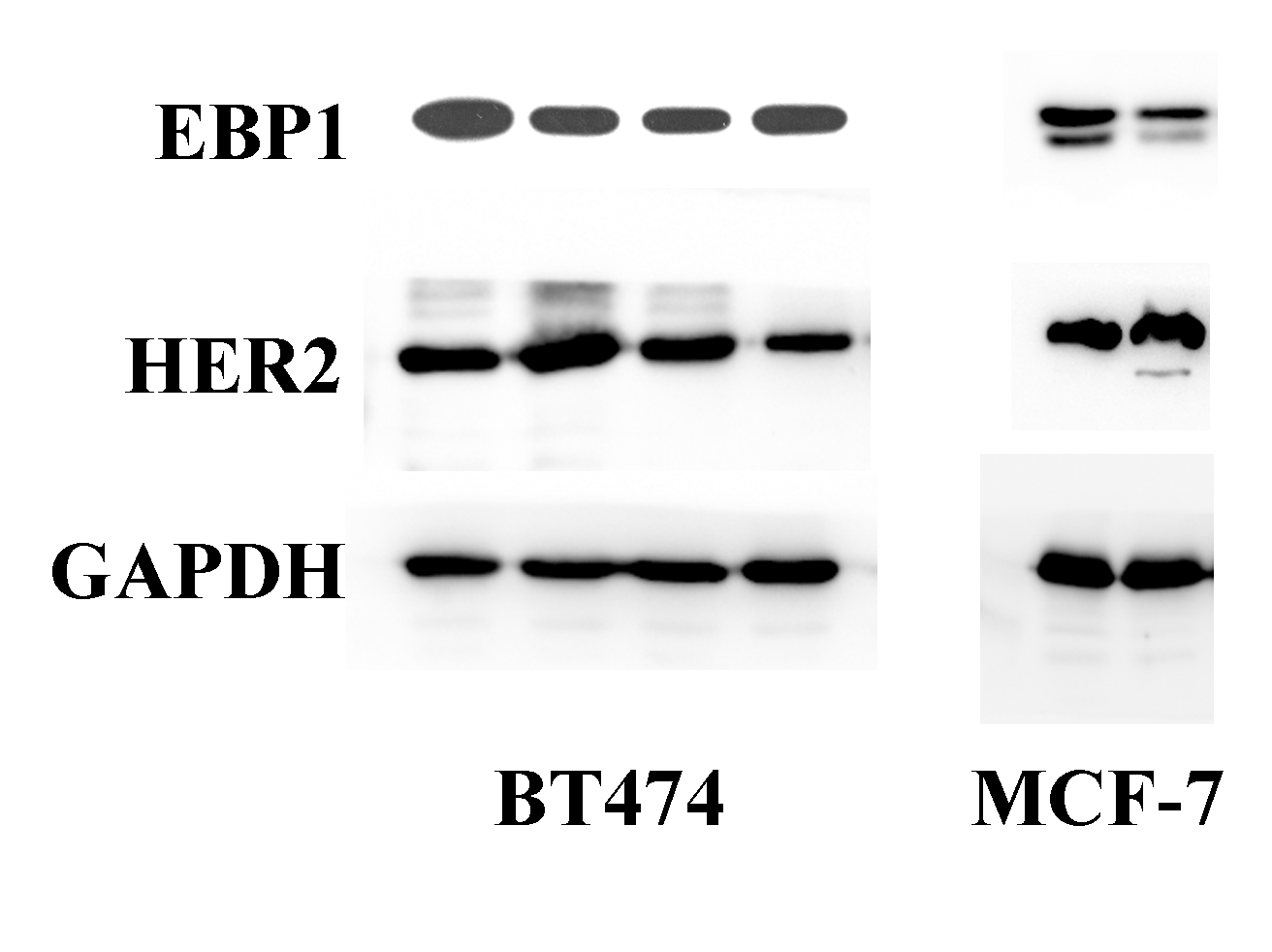

Supplement: S3 Fig — (TIF) [file pone.0245832.s003.tif]

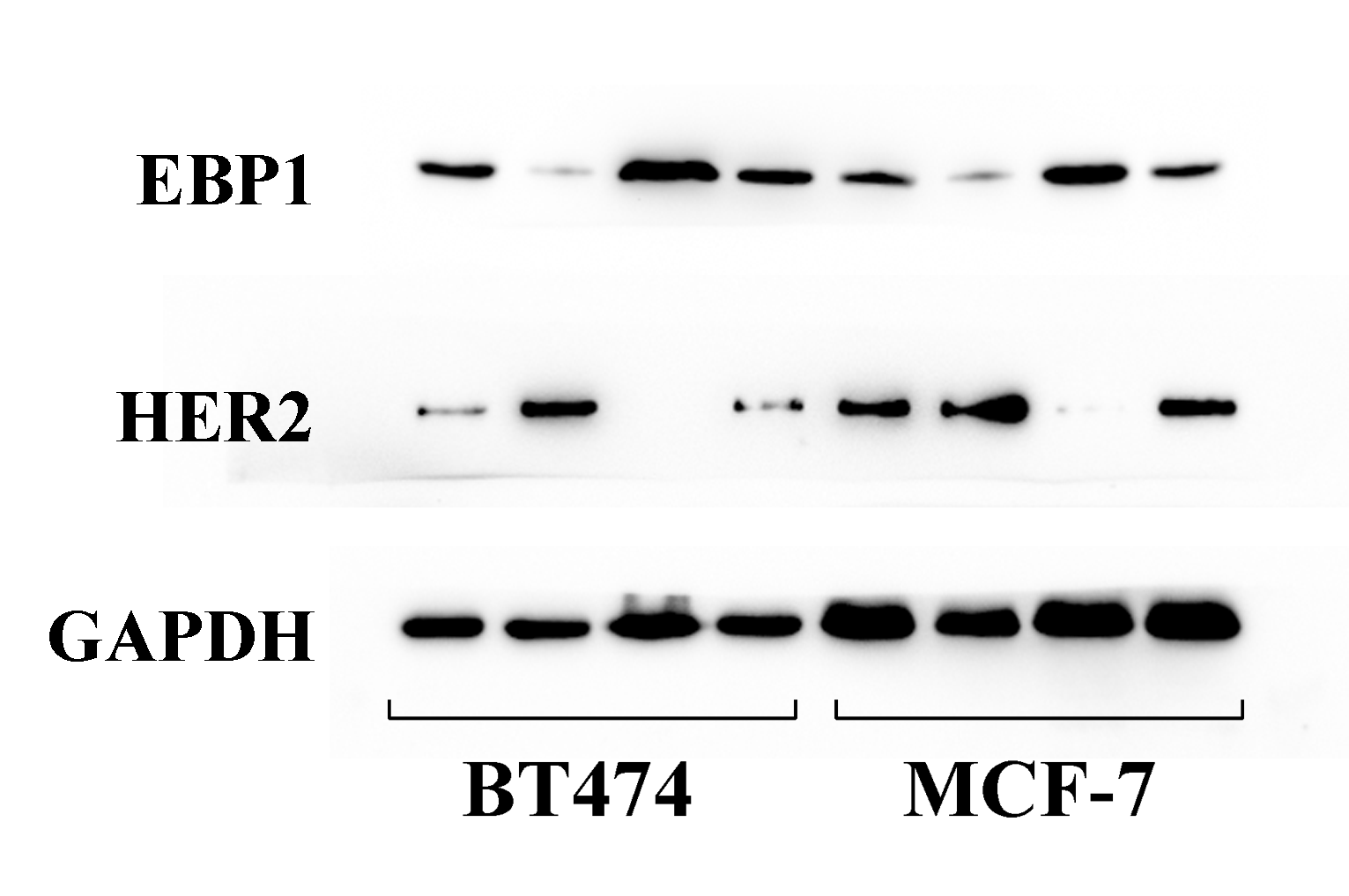

Supplement: S4 Fig — (TIF) [file pone.0245832.s004.tif]
