# Supplementary material for: HER2-intronic miR-4728-5p facilitates HER2 expression and accelerates cell proliferation and migration by targeting EBP1 in breast cancer
Source: PLoS One. 2021 Feb 2;16(2):e0245832. doi: 10.1371/journal.pone.0245832 (PMC7853520; doi:10.1371/journal.pone.0245832)
Supplement: S1 Table — (DOCX) [file pone.0245832.s005.docx]

**S1 Table.** The detailed information for the analysis of survival outcomes with HER2 and miR-4728 expression.

| Fig 1A | Fig 1B |
| --- | --- |
| **Affy ID: 210930_s_at HER2**  **Survival: OS**  Auto select best cutoff: checked  Follow up threshold: all  Censore at threshold: checked  Compute median over entire database: false | **miRNA ID: hsa-miR-4728**  **Survival:** **OS**  Auto select best cutoff: checked  Follow up threshold: all  Censore at threshold: checked  Dataset: TCGA |
| **Cutoff value** used in analysis: 116  Expression range of the probe: 2 - 10173 | **Cutoff value** used in analysis: 3  Expression range of the probe: 0 - 166 |
| **Restrictions**  ER status: all  PR status: all  HER2 status: all  Intrinsic subtype: all  Lymph node status: all  Grade: all  TP53 status: all  Pietenpol subtype: all | **Restrictions**  IHC: all  Gene chip: all  IHC: all  IHC: all  Gene chip: all  Molecular subtype: all  Grade: all  Lymph node status: all |
| **Quality control**  Remove redundant samples: checked  Array quality control: exclude biased arrays  Proportional hazards assumption: 0 |  |
| **Cohort**  Cohorts: patients with following systemic treatment  Endocrine therapy: any  Chemotherapy: any | **Cohort**  Cohorts: systemically treated patients  ER-positive endocrine therapy: any  Chemotherapy: any |
| **Results -** P value: 0.0127 | **Results -** P value: 0.0402 |
| mRNA Database [16] | miRNA Database [17] |
